# Laparoscopic Repair of a Large Incarcerated Intraparietal Inguinal Hernia Mimicking Acute Appendicitis: A Case Report

**DOI:** 10.7759/cureus.60448

**Published:** 2024-05-16

**Authors:** Lamyaa Althawadi, Nour Elddin F. Alshaer, Abdulrahman Senjab, Mohamed Hajjar

**Affiliations:** 1 General Surgery, Specialized Medical Center, Riyadh, SAU; 2 Medicine, Al-Faisal University, Riyadh, SAU

**Keywords:** incarcerated intraparietal hernia, laparoscopic tapp repair, hernia, interparietal, inguinal

## Abstract

Inguinal hernia is common. However, only a few cases have been reported in the literature of inguinal interparietal hernia, in which the herniated sac exits the intraperitoneal cavity through the deep inguinal ring and then protrudes into one of the anatomical planes of the anterior abdominal wall. Only one of the reported inguinal interparietal hernia cases was managed laparoscopically. We report the case of a right inguinal interparietal hernia in a young, healthy adult with a herniated cecum, terminal ileum, and appendix. He presented with right iliac fossa pain. On investigation, he was found to have an interparietal hernia in the inguinal region. Diagnostic laparoscopy showed a clear herniated cecum through the internal inguinal ring into the anterior abdominal wall. The patient was treated with regular laparoscopic hernioplasty and was discharged on the second postoperative day with no complications. Although the diagnosis might be difficult for interparietal hernias, laparoscopic diagnosis and management provide precise assessment and less invasive management.

## Introduction

Approximately 75% of all hernias occur in the inguinal region, making the inguinal hernia the most common type [1]. An interparietal hernia is when herniated intraperitoneal content appears through a defect in the abdominal wall and into one of the anterior abdominal wall anatomical planes [2]. It was first described in 1661 by Thomas Bartholin [3]. Interparietal hernia, like Spigelian, is a well-established rare type of hernia; it accounts for 0.12% to 2% of all hernias [4]. A Spigelian hernia is defined as a herniated sac through a defect in the Spigelian fascia and the protrusion of the sac into one of the abdominal wall layers [1,5]. Around 90% of Spigelian hernias occur below the level of the umbilicus [1]. Interparietal hernias were classified by Lower and Hicken in 1931 based on the anatomical plane of the abdominal wall [4].

The majority of interstitial interparietal hernias were attributed to incisional defects following laparotomies, or a few cases of laparoscopic port site hernia [2,4,6,7]. On the contrary, very few cases of interparietal hernias were reported in patients with no previous abdominal surgeries, most of which are located in the inguinal region [3,5,8-13]. The patient's clinical presentation varies from vague pain and discomfort at the hernia site, to obstructive and strangulation symptoms. Although the CT scan is a sensitive modality to diagnose abdominal wall hernias, it could fail to differentiate between Spigelian and inguinal interparietal hernias due to their similar anatomical location [5,12]. Hence, the final diagnosis is accomplished intraoperatively in all cases. All of the reviewed cases were managed with conventional open surgical exploration and repair [3,5,9-13] except for one case, which was successfully managed laparoscopically [8].

We report the case of a rare variant of inguinal hernia: an interstitial inguinal interparietal hernia mimicking appendicitis clinically with intact hernia orifices. This was in addition to the radiological finding of high-grade bowel obstruction due to ileocecal incarceration in a previously healthy gentleman.

## Case presentation

A 44-year-old gentleman with no significant medical or surgical history presented to the emergency department with a three-day history of abdominal pain starting at the epigastric and periumbilical areas, which then started to be localized to the right iliac fossa. The pain was dull, aching, progressive in intensity, and associated with nausea and anorexia. However, there was no associated alteration in bowel habits and no history of vomiting or fever. 

**Figure 1:**
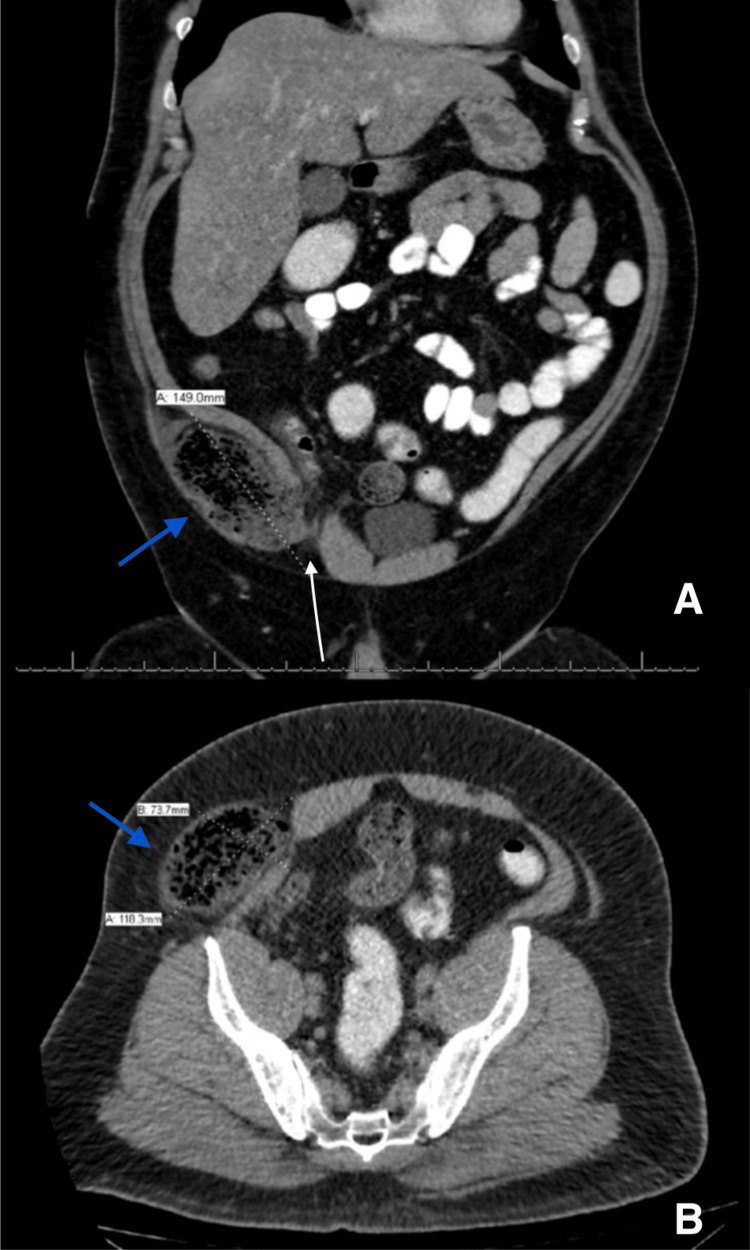
CT of the abdomen A: Coronal cut of the abdominal CT scan showing the incarcerated wall of the cecum, ileum, and appendix (blue arrow points to the herniated bowel) in relation to the inguinal region(white arrow points to the abdominal wall defect); B: Axial cut with similar findings

On physical examination, he was afebrile, and his BMI was 46 kg/m2. The abdomen was soft and lax, with tenderness and rebound tenderness on the right iliac fossa, which raised the suspicion of acute appendicitis given his clinical presentation. On investigation, the complete blood count was unremarkable. A CT scan of the abdomen performed with intravenous, oral, and rectal contrast showed a right lower abdominal wall hernia sized 11.8 x 7.3 x 14.9 cm, seen lateral to the right rectus muscle. Inferiorly, it was close to the inguinal canal with superior extension at the level of the iliac crest, containing parts of the terminal ileum, the ileocecal valve, and the base of the cecum (Figure 1).

**Figure 2:**
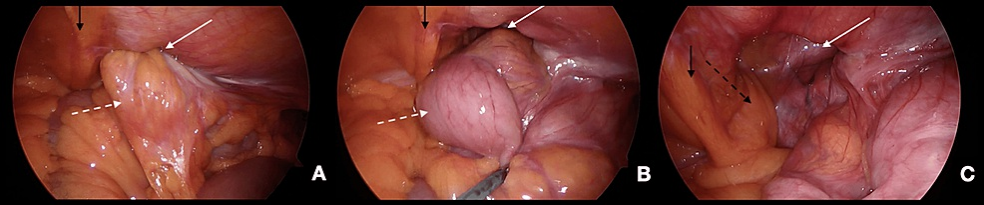
Intraoperative findings A: Herniated cecum, appendix, and terminal ileum (dashed white arrow), large defect identified in the inguinal region (white arrow) B: The reduction of herniated healthy-looking bowel (dashed white arrow) C: The hernia sac is seen through the dilated deep inguinal ring (white arrow), lateral to the inferior epigastric vessels, and spermatic cord (dashed black arrow). The black arrow in A, B, and C points to the medial umbilical fold.

Herniated bowels were distended and loaded with fecal matter, with no passage of the oral contrast and a collapsed distal colon. The appendix was hardly seen without obvious signs of appendicitis. Due to the CT findings of the incarcerated, potentially strangulated ventral hernia, the patient was taken to the operating room and underwent a diagnostic laparoscopy with hernia repair. During surgery, a large defect was seen through the deep inguinal ring, with a herniated cecum, appendix, ileocecal valve, and terminal ileum (Figure 2).

Hernia content was reduced and examined, and due to the healthy, viable bowel, no resection was required. After hernia reduction, the defect was seen lateral to the inferior epigastric vessels, and the spermatic cord was identified on the medial edge of the defect. Transabdominal preperitoneal (TAPP) hernia mesh repair was done with no complications. The patient improved after the surgery and was discharged on the second postoperative day in good condition.

## Discussion

**Figure 3:**
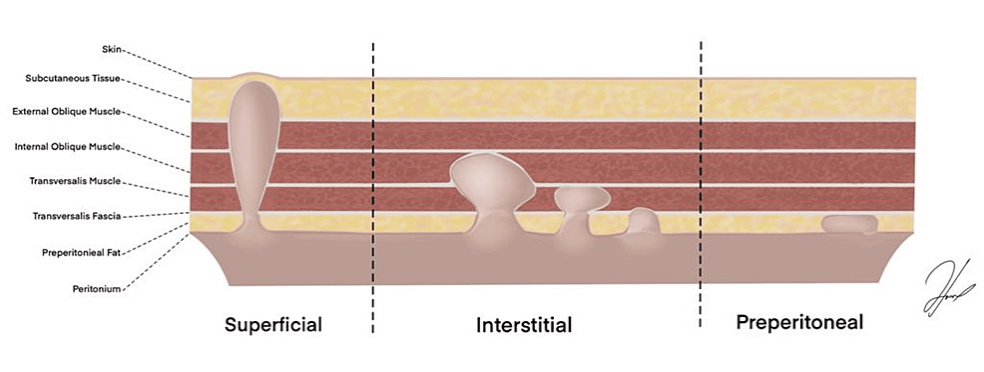
Types of interparietal inguinal hernias in relation to the abdominal wall structures Interparietal hernias classification per Lower and Hicken [4] illustrated by author Alshaaer NEF.

Interparietal hernia is a rare type of hernia, with an incidence of 0.01% to 1.6%, as reported by Lower and Hicken in the largest case series. And it has been classified into three subtypes: superficial, interstitial, and properitoneal (Figure 3) [4]. The interstitial subtype is considered the most frequently encountered of the three subtypes and accounts for 60% of all interparietal hernias [3]. The most common finding of all cases reported previously in patients with no previous abdominal surgeries is the location in the inguinal region. Due to the rarity of the hernia, no clear etiology has been defined in the literature yet [10].

Our patient demonstrated clinical symptoms of acute appendicitis while lacking obstructive symptoms and a fever. On examination, tenderness at the right iliac fossa was encountered with fullness rather than a palpated hernia. The patient's BMI is 46 kg/m2, with central obesity; however, despite his BMI during the examination, his hernial orifices were intact. A CT abdomen was requested to rule out acute, complicated appendicitis. The imaging proposed a diagnosis of Spigelian hernia due to the site of the identified defect located at the lateral edge of the rectus abdominis muscle, in addition to the interparietal protrusion of the herniated content between the external and internal oblique muscles. To our knowledge, this is the first case of inguinal interparietal hernia resembling acute appendicitis.

In our case, diagnostic laparoscopy demonstrated the hernia defect to be at the deep inguinal ring lateral to the inferior epigastric vessels. The laparoscopic TAPP mesh repair in this patient was successful, with similar outcomes in the literature. The only case reported with laparoscopic management was for an obstructing interparietal inguinal hernia with an incarcerated small bowel.

An inguinal interparietal hernia is difficult to distinguish from a Spigelian hernia on a clinical and radiological basis alone [8]. Therefore, we believe diagnostic laparoscopy is the best modality to confirm the diagnosis. In addition, the inguinal interparietal hernia type can be repaired safely with the same principles as conventional inguinal TAPP repair. However, the ultimate surgical plan and choice of operation rely heavily on the surgeon's experience and the patient’s factors.

## Conclusions

An inguinal interparietal hernia is a very rare entity. It is a challenging diagnosis, even with diagnostic imaging. However, the confirmatory diagnosis and management can be accomplished successively and safely through minimally invasive laparoscopic surgery.
